# Long-term survival rates of patients with stage IIIB and IV non-small cell lung cancer treated with cisplatin plus vinorelbine or gemcitabine

**DOI:** 10.3892/etm.2012.714

**Published:** 2012-09-18

**Authors:** SEVKET OZKAYA, SERHAT FINDIK, ADEM DIRICAN, ATILLA GÜVEN ATICI

**Affiliations:** 1 Department of Pulmonary Medicine, Faculty of Medicine, Rize University, Rize;; 2 Department of Pulmonary Medicine, Faculty of Medicine, Ondokuz Mayis University, Samsun;; 3 Department of Pulmonary Medicine, Samsun Medical Park Hospital, Samsun, Turkey

**Keywords:** non-small cell lung cancer, long-term survival, cisplatin, vinorelbine, gemcitabine

## Abstract

Limited data exist concerning the long-term (≥5 year) survival rates of patients with stage IIIB and IV non-small cell lung carcinoma (NSCLC) receiving chemotherapy. We aimed to determine the long-term results of cisplatin plus third-generation (vinorelbine or gemcitabine) cytotoxic chemotherapy in patients with locally advanced and advanced NSCLC. The study included 141 patients, and all patients were followed up from the time of diagnosis until death. The median age of the patients was 59.1±9.9 years. The male-to-female ratio was 124/17; 62.4% of the patients had stage IIIB and 37.6% had stage IV NSCLC. Squamous cell carcinoma, adenocarcinoma and undifferentiated NSCLC subtypes accounted for 69.5, 17.7 and 12.7% of the cases, respectively. The overall response rate was 32.6% and the median survival time was 12.3 months (95% CI, 10.2–14.5). The median survival times for stages IIIB and IV were 12.6±1.4 and 11.9±1.7 months, respectively. The 1-, 2-, 3- and 5-year survival rates were 33, 7.5, 4.3 and 2.8%, respectively. In conclusion, cisplatin-based new-generation cytotoxic agents for combined modality therapy offer an increased hope of long-term survival for patients with locally advanced and advanced NSCLC.

## Introduction

Lung cancer is the leading cause of cancer-related death in the world, and non-small cell lung cancer (NSCLC) accounts for 80–85% of lung cancer cases. Patients with early-stage NSCLC have relatively high long-term survival rates after surgical resection, but a substantial majority of patients, ∼80%, present in advanced or metastatic stages. Over the past decade, third-generation agents such as vinorelbine, taxanes and gemcitabine have been introduced for the treatment of NSCLC (1). Combination of one or more of these agents with a platinum compound has resulted in high response rates and prolonged overall survival. Today, doublet chemotherapies consisting of platinum plus one of the third-generation agents have become the current standard regimen, the first line of chemotherapy (2). Many studies have examined short-term survival rates in patients receiving such treatment, yet current evidence regarding long-term survival in advanced-stage NSCLC, particularly in stages IIIB and IV, is limited. This study aimed to determine the long-term results of cisplatin plus third-generation (vinorelbine or gemcitabine) cytotoxic chemotherapy in patients with locally advanced and advanced NSCLC.

## Materials and methods

### Patients

The patients with stage IIIB and IV NSCLC were evaluated at the Department of Pulmonary Medicine, Ondokuz Mayis University's Faculty of Medicine, between January 2001 and September 2004. The patient data, which included demographic, clinical, radiological, disease characteristics and therapy regimens were retrospectively obtained from the files of the patients in departmental archives. We followed up the patients from the time of diagnosis until death, and all of the patients had succumbed to causes related to lung cancer. A total of 196 patients were enrolled in the study, however, 55 (28%) were excluded due to factors rendering them inappropriate for the study and thus 141 patients were included. Approval from the patients and the institution was obtained in order to use their records for our study.

### Eligibility criteria

Performance status was classified in accordance with the criteria of the European Cooperative Oncology Group (ECOG). Staging was conducted by evaluation of imaging methods, chest X-ray, thoracic computed tomography, abdominal computed tomography, abdominal ultrasonography (USG), cranial computed tomography and bone scintigraphy. The criteria for eligibility included pathologically confirmed NSCLC, radiologically measurable lesions, an age of at least 18 years, adequate hematological function (as indicated by a white cell count of at least 4,000/ml^3^ and a platelet count of at least 100,000/ml^3^), hepatic function (as indicated by a bilirubin level that did not exceed 1.5 mg/dl, and by AST and ALT levels being <3 times the normal values), renal function (as indicated by a creatinine level that did not exceed 1.5 mg/dl), and ECOG performance status (PS) ≤2. Criteria for exclusion from the study were as follows: insufficient hematological, renal or hepatic functions; unstable brain metastasis; history of prior chemotherapy and/or radiotherapy; presence of uncontrolled infections; presence of an additional malignancy; presence of a systemic disease contradicting administration of chemotherapy; pregnancy; ECOG PS >3; and unfitness for follow-up due to psychological, familial, sociological or geographical reasons.

### Treatment plan

The main treatment regimen consisted of chemotherapy for patients in stage IV of the disease, and sequential chemoradiotherapy was used for patients in stage IIIB. Vinorelbine at a dose of 30 mg/m^2^ or gemcitabine at a dose of 1,250 mg/m^2^ on Days 1 and 8 and cisplatin at a dose of 80 mg/m^2^ on Day 1 were administered on a three-week cycle. The cycle was repeated every three weeks. At least two cycles were administered to the patients who were considered assessable for response. Patients who responded to the treatment and did not show signs of toxicity or progression received four to six cycles. Dosage was adjusted according to hematological, neurological, renal and hepatic functions. Dosage was decreased by 25% for patients who were classified as Grade III or Grade IV in accordance with WHO toxicity criteria. Curative radiotherapy was administered to all patients in stage IIIB of the disease who responded to chemotherapy after three cycles of chemotherapy regimens. A final one to three cycles were administered between three weeks and one month after administration of radiotherapy. Standard ECOG response criteria were used. The response was evaluated by thorax CT scan after two cycles of chemotherapy and at the end of the treatment. Briefly, a complete response was defined as the absence of disease at all known sites for at least four weeks. A partial response was defined as a 50% reduction in the sum of the perpendicular diameters of all measurable lesions, lasting at least four weeks. Progressive disease was defined as either a 25% increase in the area of any one lesion over the prior measurement or the development of one or more new lesions. Survival was calculated from the date of diagnosis until the date of death.

### Statistical analysis

Data were evaluated with SPSS 13.0 (SPSS, Inc., Chicago, IL, USA). Survival of the patients was calculated from the date of diagnosis to the date of death. Response rates were calculated for patients with complete or partial responses. Median age, smoking habits, performance status, response rates and toxicity results of the groups were compared with the Mann-Whitney U and Pearson Chi-square tests. The survival rates were calculated by the Kaplan-Meier method.

## Results

**Figure 1:**
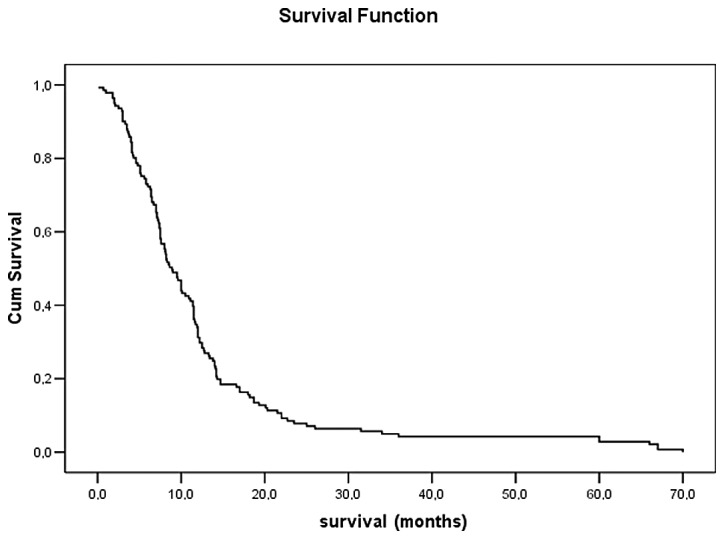
Kaplan-Meier survival curve of all patients.

**Figure 2:**
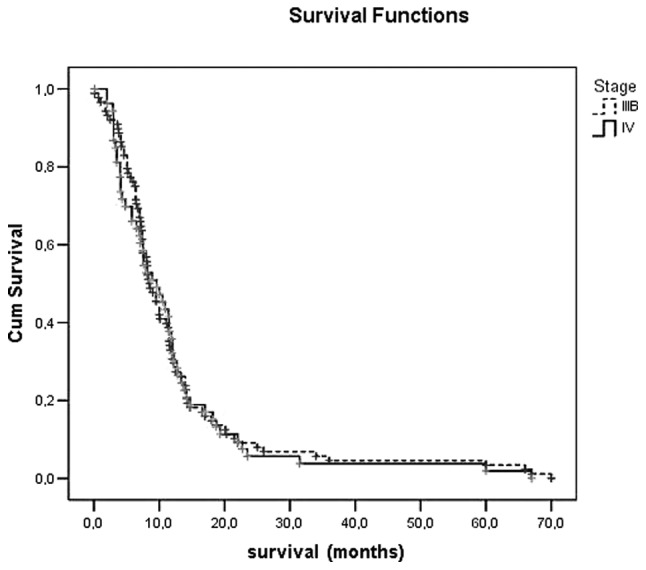
Kaplan-Meier survival curves of patients with stage III and IV.

The median age was 59.1±9.9 years and the male-to-female ratio was 124/17. Most of the patients had smoked >21 packs/year (81.6%). In the distribution of ECOG PS, 46.1% of patients were in ECOG 0–1, 43.3% of patients in ECOG 2 and 10.6% of patients in ECOG 3–4. It was observed that 62.4% of the patients had stage IIIB disease and 37.6% of patients had stage IV disease. Furthermore, 69.6% of patients had squamous cell carcinoma, 17.7% of patients had adenocarcinoma and 12.7% of patients were classified as having undifferentiated NSCLC. The metastatic sites were bone (15%), brain (10.5%), liver (6%) and adrenal gland (4.5%) in the metastatic patients (Table I). The median number of chemotherapy cycles was 3.7 for cisplatin plus third-generation (vinorelbine or gemcitabine) agents. The overall response rate was 32.6%. Respectively, 32.9 and 18% of patients received curative and palliative radiotherapy, and 13.5% of patients received second-line chemotherapy. The median survival time was 12.3 months (95% CI, 10.2–14.5). The median survival times for stages IIIB and IV were 12.6±1.4 and 11.9±1.7 months, respectively. The 1-, 2-, 3- and 5-year survival rates were 33, 7.5, 4.3 and 2.8%, respectively (Table II). All patients were evaluated for toxicity. The major hematological toxicities encountered in this study were neutropenia, febrile neutropenia, thrombocytopenia and anemia. Percentages of grades 1–2 and 3–4 toxicity for anemia were 43.9 and 8.1%, for neutropenia 40.2 and 9.6%, for thrombocytopenia 8.9 and 5.1% and for nausea and vomiting 61.9 and 3.6%, respectively. The rate of febrile neutropenia was 4.9% (Table III). No serious hemorrhagic events were noted with either regimen. Kaplan-Meier survival curves of all patients, stage III and IV are shown in Figs. 1 and 2.

## Discussion

According to reported studies, the long-term survival rate of patients with locally advanced and advanced NSCLC, varies by disease characteristics but is generally low, with 5-year survival rates for all stages ranging from 9 to 15%. The reported survival rates in stages IIIA (14.1%), IIIB (4.6%) and IV (4.2%) are closely comparable to those reported from other centers for IIIA (8–11%), IIIB (1–5%) and IV (1–5%) (3–5).

Hagerty *et al* (6) emphasized the importance of patient preferences in the case of metastatic disease from a variety of cancers, with patients more frequently wanting to know the longest survival time with treatment rather than the 5-year survival rate. However, data are sparse concerning the 5-year survival rates and 5-year survival advantages of patients with stage IIIB and IV NSCLC who receive chemotherapy or chemoradiotherapy.

Okamoto *et al* (7) reported that 7.7% of 222 metastatic NSCLC patients survived for >2 years. Satoh *et al* (8) reported that 19.4 and 13.9% of advanced NSCLC patients survived for >2 or 3 years with cisplatin-based chemotherapy, respectively. In this study, all patients received platinum-based chemotherapy as a first-line chemotherapy, and the response rate was found to be 42.8%. Kaira *et al* (9) reported a 20% response rate with first-line chemotherapy and 8% of patients survived for >5 years. These findings are similar to the findings of our study. Wang *et al* (5) evaluated 56 patients with stage III and IV NSCLC who had survived for 5 years or longer. Only one (1.7%) patient with stage IV NSCLC survived for 5 years treated with chemotherapy alone.

There is a wide variety in the toxicity rates reported in previous studies, with grade 3–4 anemia in 7–24% and 20–30% of the patients receiving cisplatin and vinorelbine and the patients receiving cisplatin and gemcitabine, respectively; grade 3–4 neutropenia in 5.4–38.5% and 13.8–81%; grade 3–4 thrombocytopenia in 2.5–20% and 2.5–6%; and grade 3–4 nausea and vomiting in 0–58% and 3.2–39%, respectively (10–13). Our toxicity results were consistent with those of previous studies.

In conclusion, the effects of chemotherapy in advanced-stage NSCLC patients have been controversial since the 1990s. However, the cisplatin-based new-generation cytotoxic agents for combined modality therapy offer increased hope of long-term survival of patients with locally advanced and advanced NSCLC. There is a continued need to follow up the outcomes of such patients over long periods of time.

## Figures and Tables

**Table I:** Baseline characteristics of the patients.

Characteristics	n=141
Age (median ± SD)	59.1±9.9
Gender (%)	
Male	87.9
Female	12.1
Smoking status (%)	
Nonsmoker	5.0
<10 packs/year	5.0
10–20 packs/year	8.5
21–30 packs/year	20.6
>30 packs/year	61.0
ECOG performance status (%)	
0–1	46.1
2	43.3
3–4	10.6
Disease stage (%)	
IIIB	62.4
IV	37.6
Histological type (%)	
Squamous cell	69.5
Adenocarcinoma	17.7
Undifferentiated NSCLC	12.7
Metastatic sites (%)	
Bone	15.0
Brain	10.5
Liver	6.0
Adrenal	4.5

**Table II:** Outcomes of the treatments.

Variables	n=141
Response (%)	
Complete response	6.8
Partial response	26.1
Stable disease	40.5
Progressive disease	26.6
Overall response rate (%)	32.6
Radiotherapy (%)	
Curative	32.9
Palliative	18.0
Second-line chemotherapy (%)	13.5
Overall survival [months (95% CI)]	12.3 (10.2–14.5)
Stage IIIB median survival (months)	12.6±1.4
Stage IV median survival (months)	11.9±1.7
Survival (%)	
1-year survival	33.0
2-year survival	7.5
3-year survival	4.3
5-year survival	2.8

**Table III:** Toxic effects.

Type of toxicity	n=141
Anemia (%)	
Grade 1–2	43.9
Grade 3–4	8.1
Neutropenia (%)	
Grade 1–2	40.2
Grade 3–4	9.6
Febrile neutropenia (%)	4.9
Thrombocytopenia (%)	
Grade 1–2	8.9
Grade 3–4	5.1
Nausea and vomiting (%)	
Grade 1–2	61.9
Grade 3–4	3.6
